# The effect of isocaloric macronutrient compositions on hepatic cellular senescence in aging mice

**DOI:** 10.1093/geroni/igaf122.1925

**Published:** 2025-12-31

**Authors:** Samantha Jezak, Tamara Pulpitel, Amanda Brandon, Stephen Simpson, Christopher Wiley

**Affiliations:** Tufts University, Medford, Massachusetts, United States; The University of Sydney, Sydney, New South Wales, Australia; The University of Sydney, Sydney, New South Wales, Australia; The University of Sydney, Sydney, New South Wales, Australia; Jean Mayer HNRCA at Tufts University, Boston, Massachusetts, United States

## Abstract

Macronutrients (proteins, carbohydrates, and fat), independent of caloric intake, impact health span (HS) and lifespan (LS) in mice. A low-protein: high-carbohydrate diet yields the longest lifespans in both male and female mice. The cellular mechanisms responsible for mediating this relationship have not been thoroughly investigated, but cellular senescence may play an important role. In response to stress and upon natural aging, cellular senescence manifests as an essentially irreversible cell cycle arrest, accompanied by an often pro-inflammatory senescence-associated secretory phenotype (SASP), which damages the surrounding environment and contributes to accelerated aging and degenerative pathology. To better understand how isocaloric macronutrient compositions affect hepatic cellular senescence in aging mice, we measured markers of cellular senescence in the liver at four different timepoints in males and females fed 20 different diets. To quantify the synergistic impact of all three macronutrients, we used the Geometric Framework for Nutrition (GFN) – a multi-dimensional modeling system that allows n-nutrients to be considered for a particular outcome. Females had increased expression of all cell cycle arrest markers compared to males. We found that three senescence cell cycle arrest markers (p16INK4a, p15INK4b, and p21WAF1) responded differently to diet. p16INK4a expression was lowest on a high-protein: high-carb: low-fat diet. However, p21WAF1 expression was lowest on a low-protein: low-carb: high-fat diet. p15INK4b was more similar to p21WAF1 but displayed less dramatic variation as macronutrient distributions changed. This data identifies macronutrients’ influence on the cell cycle arrest arm of senescence and may provide new insights on mechanisms mediating diet and aging.

